# Supplementary material for: Integrative weighted molecular network construction from transcriptomics and genome wide association data to identify shared genetic biomarkers for COPD and lung cancer
Source: PLoS One. 2022 Oct 4;17(10):e0274629. doi: 10.1371/journal.pone.0274629 (PMC9531836; doi:10.1371/journal.pone.0274629)
Supplement: S1 Fig — The PPI network at p-value >0.05 consist of 995 nodes interact with 18924 edges. (PDF) [file pone.0274629.s001.pdf]

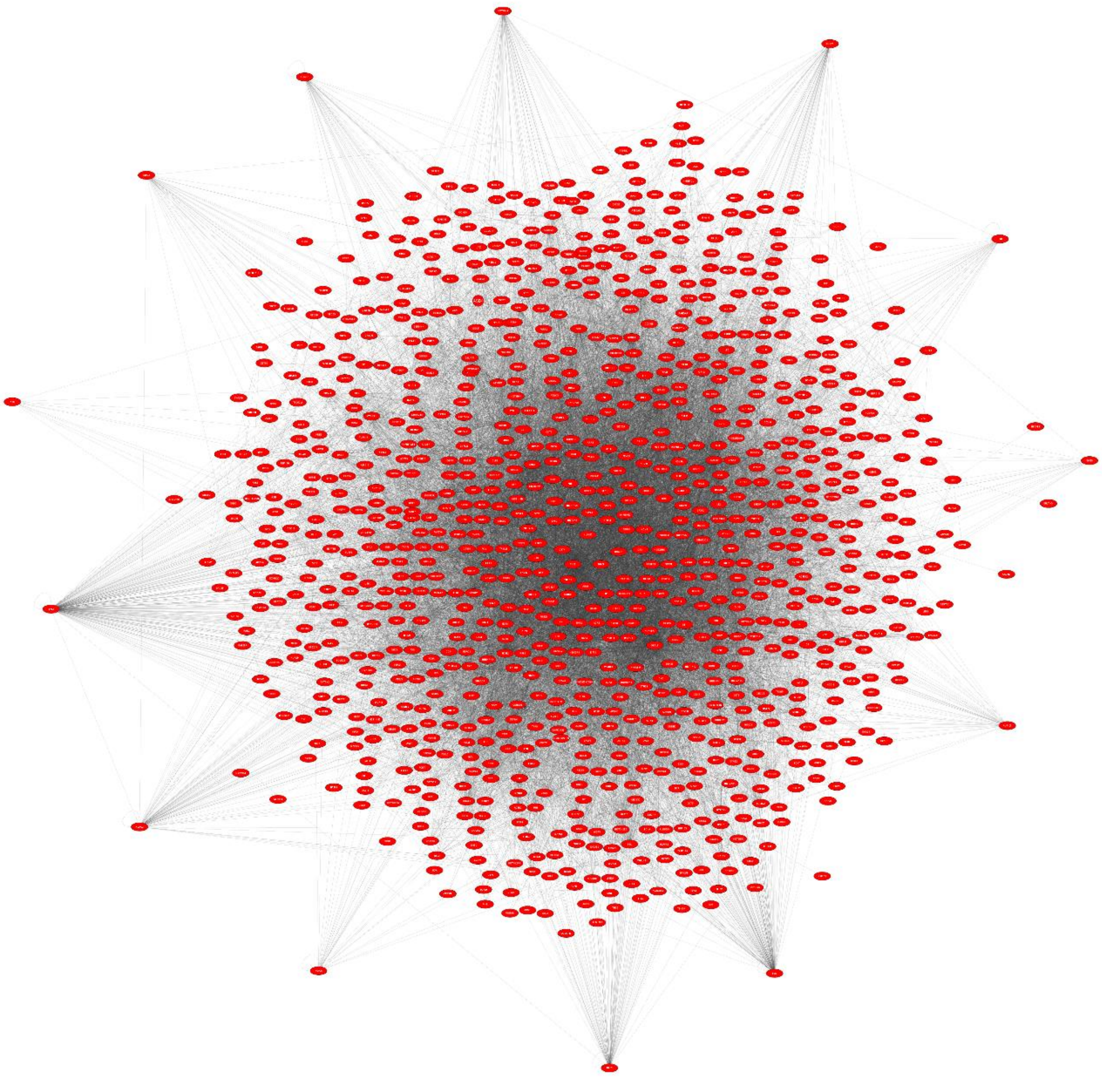

S1 Fig: Overview of PPI network constructed from 63 common genes using Cytoscape STRING database. The PPI network at p-value >0.05 consist of 995 nodes interact with 18924 edges.
