# Supplementary material for: Integrative weighted molecular network construction from transcriptomics and genome wide association data to identify shared genetic biomarkers for COPD and lung cancer
Source: PLoS One. 2022 Oct 4;17(10):e0274629. doi: 10.1371/journal.pone.0274629 (PMC9531836; doi:10.1371/journal.pone.0274629)
Supplement: S2 Fig — (A) Cluster-1 (SREK1). (B) Cluster-2 (TMEM67). (C) Cluster-3 (IRAK2). (D) Cluster-4 (MECOM). (E) Cluster-5 (ASB4). (F) Cluster-6 (CDC42BPA). (G) Cluster-7 (DPF3). (H) Cluster-8 (DET1). (I) Cluster-9 (KHK). (J) Cluster-10 (DDX3Y), the hub gene selected based on high centrality in the protein network of DEGs. (PDF) [file pone.0274629.s002.pdf]

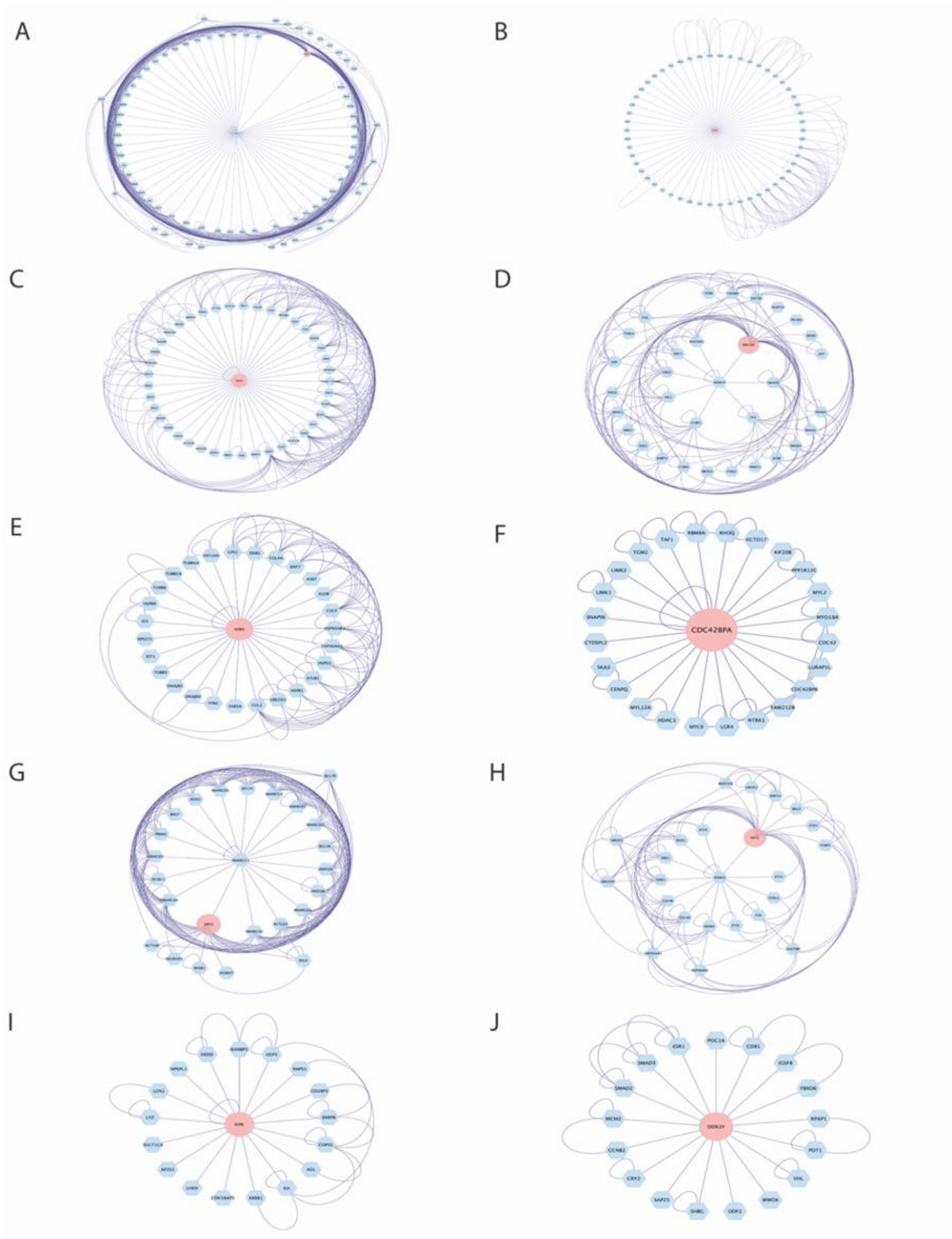

S2 Fig. The 12 hub gene protein interaction network. (A) Cluster-1 (SREK1). (B) Cluster-2 (TMEM67). (C) Cluster-3 (IRAK2). (D) Cluster-4 (MECOM). (E) Cluster-5 (ASB4). (F) Cluster-6 (CDC42BPA). (G) Cluster-7 (DPF3). (H) Cluster-8 (DET1). (I) Cluster-9 (KHK). (J) Cluster-10 (DDX3Y), the hub gene selected based on high centrality in the protein network of DEGs.
