# Supplementary material for: Integrative weighted molecular network construction from transcriptomics and genome wide association data to identify shared genetic biomarkers for COPD and lung cancer
Source: PLoS One. 2022 Oct 4;17(10):e0274629. doi: 10.1371/journal.pone.0274629 (PMC9531836; doi:10.1371/journal.pone.0274629)
Supplement: S3 Fig — Protein Pathology Atlas of 12 hug genes in normal lung and lung cancer tissues. (PDF) [file pone.0274629.s003.pdf]

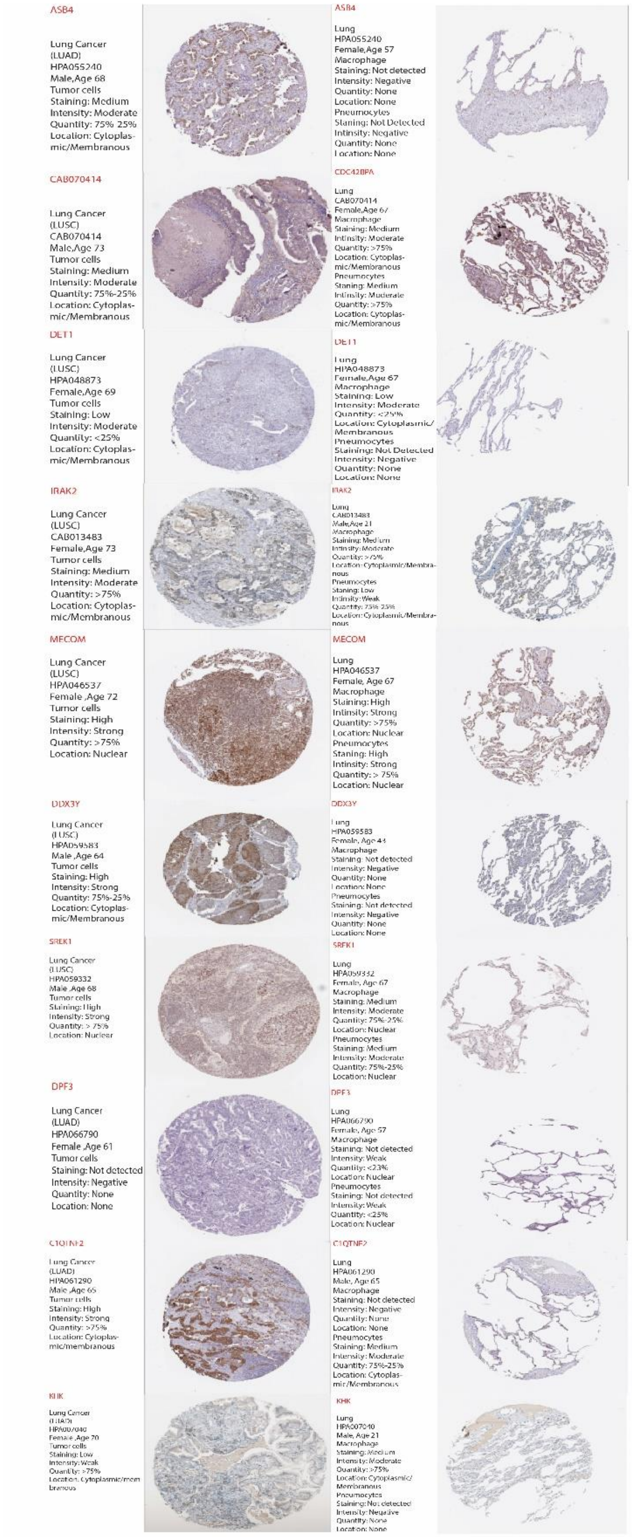

S3 Fig. Histopathological Images of DEGS. Protein Pathology Atlas of 12 hug genes in normal lung and lung cancer tissues
