# Supplementary material for: Integrative weighted molecular network construction from transcriptomics and genome wide association data to identify shared genetic biomarkers for COPD and lung cancer
Source: PLoS One. 2022 Oct 4;17(10):e0274629. doi: 10.1371/journal.pone.0274629 (PMC9531836; doi:10.1371/journal.pone.0274629)
Supplement: S1 Table — Genetic association of hub genes with the lung related traits and lung cancer from Phenoscanner database. (DOCX) [file pone.0274629.s004.docx]

**S1 Table. Hub Genes GWAS Association.** Genetic association of hub genes with the lung related traits and lung cancer from Phenoscanner database.

| **Gene** | **Variant** | **P-value** | **Trait(s)** |
| --- | --- | --- | --- |
| IRAK2 | rs544145803 | 7.72E-06 | Self-reported lung cancer |
|  | rs114743735 | 1.26E-10 | Eosinophil count |
|  | rs114743735 | 5.73E-09 | Sum eosinophil basophil counts |
|  | rs114743735 | 5.98E-11 | Eosinophil percentage of white cells |
|  | rs114743735 | 3.11E-10 | Eosinophil percentage of granulocytes |
|  | rs114743735 | 1.03E-08 | Neutrophil percentage of granulocytes |
|  | rs114743735 | 1.00E-10 | Eosinophil counts |
|  | rs114743735 | 6.00E-09 | Sum eosinophil basophil counts |
|  | rs114743735 | 6.00E-11 | Eosinophil percentage of white cells |
|  | rs114743735 | 3.00E-10 | Eosinophil percentage of granulocytes |
|  | rs4684037 | 1.95E-06 | Eosinophil count |
|  | rs4684037 | 4.77E-07 | Eosinophil percentage of white cells |
|  | rs4684037 | 4.28E-06 | Eosinophil percentage of granulocytes |
|  | rs115820364 | 7.25E-11 | Eosinophil percentage of white cells |
|  | rs115820364 | 3.61E-10 | Eosinophil percentage of granulocytes |
| MECOM | rs1362772 | 3.24E-06 | Lung function forced expiratory volume in 1 second FEV1 |
|  | rs115966694 | 1.05E-06 | lung cancer |
|  | rs139032165 | 3.76E-07 | chronic obstructive pulmonary disease with acute exacerbation, unspecified |
|  | rs79624766 | 4.81E-07 | chronic obstructive pulmonary disease with acute exacerbation, unspecified |
|  | rs180945029 | 8.01E-06 | Smoking or smokers in household |
|  | rs7642776 | 5.38E-06 | Lung function forced expiratory volume in 1 second FEV1 |
|  | rs1344555 | 2.65E-08 | Lung function forced expiratory volume in 1 second FEV1 |
|  | rs1344555 | 5.13E-06 | Lung function forced expiratory volume in 1 second FEV1 in ever smokers |
|  | rs1344555 | 3.75E-06 | Lung function forced expiratory volume in 1 second FEV1 smoking pack years |
|  | rs1344555 | 3.00E-08 | Pulmonary function |
|  | rs1344555 | 4.00E-06 | Pulmonary function interaction |
|  | rs552372355 | 3.18E-06 | Cause of death: chronic obstructive pulmonary disease with acute exacerbation, unspecified |
| CDC42BPA | rs16846779 | 4.16E-08 | Cause of death: chronic obstructive pulmonary disease with acute exacerbation, unspecified |
|  | rs151284662 | 1.89E-06 | Cause of death: chronic obstructive pulmonary disease with acute exacerbation, unspecified |
|  | rs537880461 | 1.89E-07 | Cause of death: chronic obstructive pulmonary disease with acute exacerbation, unspecified |
|  | rs185516257 | 1.40E-06 | Cause of death: chronic obstructive pulmonary disease, unspecified |
|  | rs550583008 | 6.81E-17 | Cause of death: chronic obstructive pulmonary disease with acute exacerbation, unspecified |
|  | rs184075990 | 1.41E-06 | Cause of death: chronic obstructive pulmonary disease with acute lower respiratory infection |
|  | rs537085725 | 1.08E-06 | Cause of death: chronic obstructive pulmonary disease, unspecified |
| ASB4 | rs189898348 | 5.47E-06 | Cause of death: chronic obstructive pulmonary disease with acute lower respiratory infection |
|  | rs186134857 | 2.89E-07 | Cause of death: chronic obstructive pulmonary disease with acute lower respiratory infection |
| DPF3 | rs564024069 | 2.63E-06 | Cause of death: chronic obstructive pulmonary disease, unspecified |
|  | rs549157500 | 4.09E-06 | Cause of death: chronic obstructive pulmonary disease, unspecified |
|  | rs982972 | 6.44E-06 | Lung cancer squamous cell carcinoma |
| TMEM67 | rs746297334 | 3.52E-06 | lung cancer |
