# Supplementary material for: Integrative weighted molecular network construction from transcriptomics and genome wide association data to identify shared genetic biomarkers for COPD and lung cancer
Source: PLoS One. 2022 Oct 4;17(10):e0274629. doi: 10.1371/journal.pone.0274629 (PMC9531836; doi:10.1371/journal.pone.0274629)
Supplement: Attachment — Submitted filename: Response to Reviewers.docx [file pone.0274629.s005.docx]

1. Please ensure that your manuscript meets PLOS ONE's style requirements, including those for file naming. The PLOS ONE style templates can be found at 
<https://journals.plos.org/plosone/s/file?id=wjVg/PLOSOne_formatting_sample_main_body.pdf> and <https://journals.plos.org/plosone/s/file?id=ba62/PLOSOne_formatting_sample_title_authors_affiliations.pdf>

2. Thank you for stating the following in the Acknowledgments Section of your manuscript: 
"This project was funded by the Deanship of Scientific Research (DSR) at King Abdulaziz University, under Grant no. G-593-140-1441. The authors, therefore, acknowledge the DSR for technical and financial support."

We note that you have provided funding information. However, funding information should not appear in the Acknowledgments section or other areas of your manuscript. We will only publish funding information present in the Funding Statement section of the online submission form. 
Please remove any funding-related text from the manuscript and let us know how you would like to update your Funding Statement. Currently, your Funding Statement reads as follows: 
"Funding Author: BB
Grant Number: G-593-140-1441
Full Name of Funder: Deanship of Scientific Research (DSR), King Abdulaziz University
URL: <https://dsr.kau.edu.sa/>
Role: The funders had no role in study design, data collection and analysis, decision to publish, or preparation of the
manuscript."

Please include your amended statements within your cover letter; we will change the online submission form on your behalf.

3. Please include captions for your Supporting Information files at the end of your manuscript, and update any in-text citations to match accordingly. Please see our Supporting Information guidelines for more information: <http://journals.plos.org/plosone/s/supporting-information>.

Reviewer #1: The original article by Banaganapalli and colleagues sought to identify significant changes in COPD and lung cancer. The authors collected transcriptomics and protein-protein interaction networks, and then discovered some differentially expressed genes, that are analyzed by some bioinformatics approaches and are available in silico datasets. They further predicted these clinicopathological events and patient outcomes through the features they selected. Other comments are listed as follows
**• There are different stages of COPD to determine the severity of disease status, is there any difference among their gene expression?**

Answer: Our study aimed to identify the common biomarker between COPD and lung cancer. For this reason, we ignored the disease severity variability in our study. In this study, we collected all stages of COPD (GSE54837).

**• Some figures have poor pixels, and the font is too small.**

Answer: We increased the quality of figures

• **They did not describe the statistical algorithm and p-value of each website and panel**.

Answer: WE revised as per the reviewer suggestion

• **The number of DEGs in manuscript and figure 1B are different.**

Answer: Corrected

**• Some of the web links they provide cannot be entered**

Answer: Weblinks updated

**• There is no title of Table 4. It is also better to use scoring system to quantify and present the IHC results of these genes.**

Answer: Updated in the table 4

**Reviewer #2**: In the present study, the authors aim to identify potential biomarkers for COPD diagnosis and prognosis by analyzing the dysregulated gene expression patterns from blood and lung tissues. They identified many common DEGs from blood and lung tissue datasets and focused on 12 hub genes with DC score of >17. They further performed PPI analysis, GO analysis and GWAS to reveal the significance of 12 hub genes in COPD and lung cancer. They provided massive and valuable information about COPD and lung cancer.

Minor Points:
**1. There are many references without formal styles such as Ref #10, #12, #13, #20, #28 #31, #39 and The Ref#16 is wrongly embedded. The authors should correct them.**

Answer: All the references have been updated with reference manger software

**2. The refractive asthma should be corrected as** **refractory asthma.**

Answer: Corrected

**3. There are many inconsistencies among figures, legends and contexts such as Figure 1 & 3 and table**

Answer: Updated

**4. 12 significant genes should be with more than 17 of DC in the legends of Table 1 and Figure 2.**

Answer: Table 1 shows the degree centrality of 12 significant genes, whereas Figure 2 mentioned >18 centrality score genes

**5. Go-annotations should be corrected as GO-annotations in Figure 3.**

Answer: corrected

**6. The items in table 1 should be modified to a professional one and the style in the column of Term Description should be consistent.**

Answer: Table 1 is centrality table, all the values are consistence according to the journal style

**7. The image and staining intensity of MECOM from HPA database compared to Figure S3 were wrongly embedded and the authors should correct them.**

Answer: Figure 6 was revised and now its exactly mapping to Figure S3
